# *Toxoplasma gondii*’s Basal Complex: The Other Apicomplexan Business End Is Multifunctional

**DOI:** 10.3389/fcimb.2022.882166

**Published:** 2022-04-29

**Authors:** Marc-Jan Gubbels, David J. P. Ferguson, Sudeshna Saha, Julia D. Romano, Suyog Chavan, Vincent A. Primo, Cynthia Michaud, Isabelle Coppens, Klemens Engelberg

**Affiliations:** ^1^Department of Biology, Boston College, Chestnut Hill, MA, United States; ^2^Nuffield Department of Clinical Laboratory Science, University of Oxford John Radcliffe Hospital, Oxford, United Kingdom; ^3^Department of Biological and Medical Sciences, Faculty of Health and Life Science, Oxford Brookes University, Oxford, United Kingdom; ^4^Department of Molecular Microbiology and Immunology, Bloomberg School of Public Health, Johns Hopkins University, Baltimore, MD, United States

**Keywords:** *Toxoplasma gondii*, basal complex, MORN1, cell division, endodyogeny, bradyzoite

## Abstract

The Apicomplexa are famously named for their apical complex, a constellation of organelles at their apical end dedicated to invasion of their host cells. In contrast, at the other end of the cell, the basal complex (BC) has been overshadowed since it is much less prominent and specific functions were not immediately obvious. However, in the past decade a staggering array of functions have been associated with the BC and strides have been made in understanding its structure. Here, these collective insights are supplemented with new data to provide an overview of the understanding of the BC in *Toxoplasma gondii*. The emerging picture is that the BC is a dynamic and multifunctional complex, with a series of (putative) functions. The BC has multiple roles in cell division: it is the site where building blocks are added to the cytoskeleton scaffold; it exerts a two-step stretch and constriction mechanism as contractile ring; and it is key in organelle division. Furthermore, the BC has numerous putative roles in ‘import’, such as the recycling of mother cell remnants, the acquisition of host-derived vesicles, possibly the uptake of lipids derived from the extracellular medium, and the endocytosis of micronemal proteins. The latter process ties the BC to motility, whereas an additional role in motility is conferred by Myosin C. Furthermore, the BC acts on the assembly and/or function of the intravacuolar network, which may directly or indirectly contribute to the establishment of chronic tissue cysts. Here we provide experimental support for molecules acting in several of these processes and identify several new BC proteins critical to maintaining the cytoplasmic bridge between divided parasites. However, the dispensable nature of many BC components leaves many questions unanswered regarding its function. In conclusion, the BC in *T. gondii* is a dynamic and multifunctional structure at the posterior end of the parasite.

## Introduction

**Figure 1:**
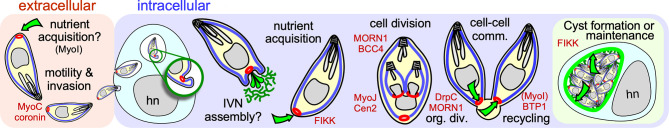
Schematic overview of established and putative roles of the BC in the intermediate host. The BC is represented in red, the IMC in blue, the IVN and cyst walls in green. Green arrows display transport through the BC. Key molecular players are shown in red at the steps where they have key functions. cell-cell comm. refers to cell-cell communication; org. div. refers to BC’s role in organization of cell division; hn marks the host cell nucleus.

The obligate intracellular Apicomplexa comprise parasites of a wide variety of animal phyla, including humans. All Apicomplexa share a universal body plan designed to invade their next host cell (Woo et al., 2015; Harding and Frischknecht, 2020). The structures dedicated to host cell invasion consist of cytoskeleton elements and secretory organelles that are concentrated on the apical end of the cell, whose distinct appearance gave the Apicomplexa their name (Cavalier-Smith and Chao, 2004). The other end of the cell, the basal complex (BC), is morphologically much less pronounced and has received much less attention. The historic appreciation for the BC revolves around its prominent role in cell division: the BC functions as the contractile ring in separating daughter parasites at the conclusion of cell division (**Figure 1**) **(** Gubbels et al., 2020). The BC is situated at the most basal extremity of the inner membrane complex (IMC), which is the apicomplexan membrane skeleton that orchestrates cell division (Gubbels et al., 2020). The IMC is part of the cortical cytoskeleton that is defined by flattened membrane vesicles (alveoli) decorated on the inside by a set of subpellicular microtubules originating at the apical end and a meshwork of intermediate filament-like proteins (alveolins or epiplastins) anchored to the membrane (Goodenough et al., 2018; Gubbels and Morrissette, 2020). The cortical cytoskeleton also anchors the myosin motors that power gliding motility and host cell invasion (Frenal et al., 2017; Gubbels and Morrissette, 2020). Indeed one of these motors, MyoB/C, resides at the basal end (Frenal et al., 2014). Furthermore, the BC maintains a cytoplasmic bridge between parasites and the residual body after division is finalized, facilitating cell-cell communication (Frenal et al., 2017). Beyond these, putative BC functions are emerging in nutrient uptake (Romano et al., 2017) and bradyzoite formation and/or maintenance (personal communication Dr. Dana Mordue; manuscript submitted) (**Figure 1**). The BC is also the site of intravacuolar network (IVN) assembly, a tubular membrane structure inside the vacuolar compartment required for access to host cell derived vesicles and establishing bradyzoite cysts, though a direct functional involvement of the BC in this process has not been established (Sibley et al., 1995). Regarding BC dynamics, discrete developmental steps in the BC can be appreciated during the division process (Anderson-White et al., 2011), whereas in extracellular parasites, the very basal end presents as a cup, i.e. the ‘posterior cup’ with a small pore (Mann and Beckers, 2001). Recent progress in defining the composition of the BC together with an expanding spectrum of functions has resulted in new insights in the molecular basis of structure-function relationships in the BC, although as discussed here, many questions remain.

## Architecture and Dynamics Throughout the Lytic Cycle

**Figure 2:**
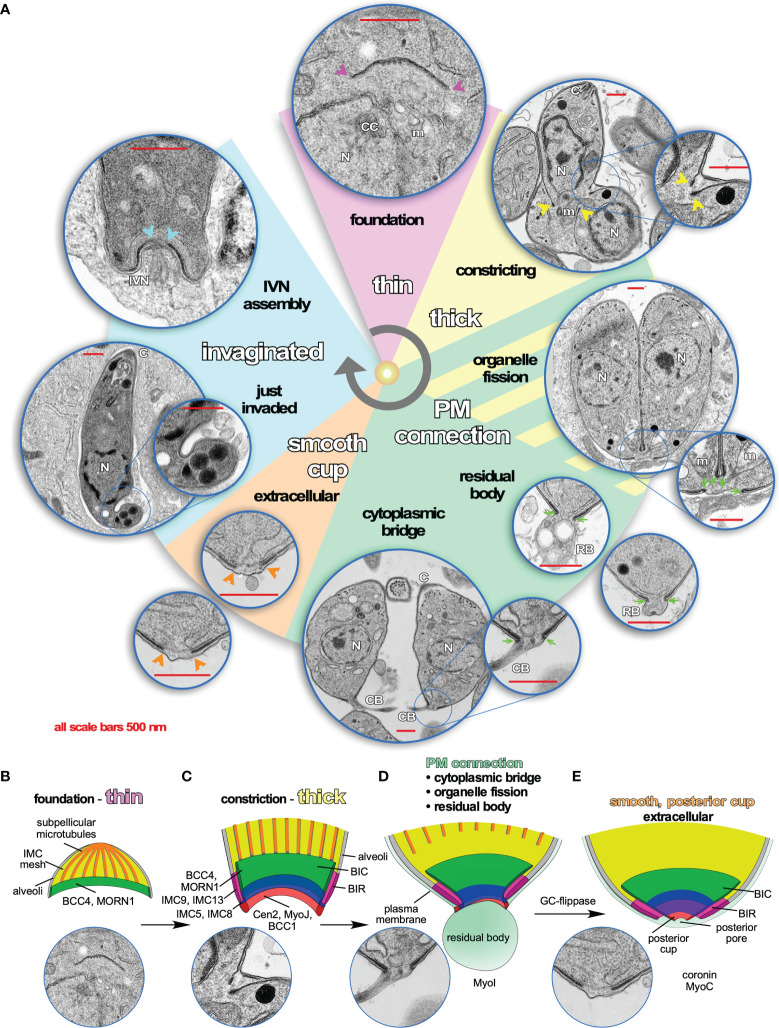
Dynamics of the Basal Complex (BC) throughout the *Toxoplasma* lytic cycle. **(A)** Pie slice colors reflect different manifestations of the BC illustrated by representative transmission electron microscopy (TEM) images. Arrows and arrowheads in the corresponding color point out the BC at critical points. Purple: during early daughter bud formation, the basal end of the forming cytoskeleton is thin, although several molecular markers of the BC are present at these early stages such as MORN1 and BCC4, which comprise the foundation for recruitment of additional markers. Yellow: when daughter development is halfway, the BC transforms into a double-layer of electron dense material, which coincides with the constriction that tapes the cytoskeleton buds toward the posterior end. Sea green: When the plasma membrane fully coats the daughter cytoskeleton, an electron-dense connection from the BC toward the plasma membrane is visible. It is postulated that this connection might be needed to partition the mitochondrion as well as to recycle material from the residual body and/or to maintain the connection with the residual body, as well as to maintain the cytoplasmic bridge connecting tachyzoites within the same vacuole that is responsible for the typical rosette-shaped tachyzoite manifestation. Orange: once the cytoplasmic bridge is broken and parasites are extracellular, the BC connection to the plasma membrane disappears and is replaced with a continuous structure on the inside of the IMC. Most likely, this is the “posterior cup” observed in detergent extracted cytoskeletons of tachyzoites (Mann and Beckers, 2001) (we note that a small hole is present in the extreme end of the detergent extracted posterior cup, which is not observed in the whole parasite TEMs shown here). Endocytosis, likely mediated by coronin that translocates to the BC in extracellular parasites, is required for efficient gliding (Salamun et al., 2014; Gras et al., 2019). Baby blue: minutes after completion of host cell invasion by tachyzoites, the basal end of the parasites has a twisted appearance. This is likely the result of tachyzoite rotations within the vacuole to pinch of the PVM (Pavlou et al., 2018). A couple of minutes later, the BC itself invaginates, still maintaining a continuous layer within the IMC, which coincides with the assembly of the IVN from the basal end. However, a direct link between BC appearance, composition or function and IVN assembly has so far not been observed and might merely be coincidental. **(B–E)** Schematic representations of the architecture and molecular organization of the first four different BC stages recognizable by TEM. **(B)** First half of daughter bud assembly. **(C)** Second half of daughter bud assembly. **(D)** BC in mature but conjoined parasites before abscission of divided cells. **(E)** BC in a fully mature, single tachyzoites (motile if extracellular).

At the ultrastructural level, the BC changes throughout *T. gondii*’s lytic cycle. We differentiate five different arrangements of the BC at the basal end of the cytoskeleton that correlate with its different functions (**Figure 2A**). During the first half of daughter cytoskeleton assembly no electron dense structure is visible at the basal end. However, we identified two proteins that are present at the BC at the very early steps in daughter cytoskeleton formation: the scaffolding protein MORN1, which is first seen as a hazy cloud surrounding the duplicated centrosomes before assembling in a ring at the initiation of each daughter cytoskeleton formation (Gubbels et al., 2006; Hu et al., 2006; Hu, 2008) and BCC4, a protein without identifiable functional domains that assembles independently from MORN1 into a ring-like structure (Engelberg et al., 2021) (**Figure 2B**). MORN1 and BCC4 are in a complex together and disruption of either protein results in incomplete daughter separation at a very severe fitness cost (Heaslip et al., 2010; Lorestani et al., 2010; Engelberg et al., 2021). These early BC proteins are essential to stabilize the growing basal ends of the daughter buds and recruit additional BC proteins. Several additional proteins associate with the BC while the cytoskeleton scaffolds are growing, including several hypothetical proteins, two phosphatases, and microtubule binding protein DIP13 (Leveque et al., 2016) (**Table 1**). Of these, the HAD2a phosphatase is essential for the parasite, and its absence presents a phenotype similar to MORN1 and BCC4 depletion (Engelberg et al., 2016), suggesting that it regulates the stability of the BCC4/MORN1 interaction.

**Table 1:** Overview of all known *T. gondii* proteins mapping to the BC. Gene IDs are derived from ToxoDB.org (Gajria et al., 2008).

#	name	TGGT1gene ID	putative function	fitness score	BCSC #	BCexcl.	timing					reference
							in	ex	co	ma	ec	
1	BCC0	294860	hypothetical	-4.10	(1)	no						(Hu et al., 2006; Engelberg et al., 2021)
2	MORN1	310440	structural filament, scaffold	-4.55	1	no						(Gubbels et al., 2006)
3	BCC4	229260	hypothetical	-3.52	1	yes						(Engelberg et al., 2021)
4	14-3-3	263090	phosphorylation signaling	-5.79	1	no						(Lorestani et al., 2012)
5	BCC3	311770	hypothetical	-0.11	2	no						(Engelberg et al., 2021)
6	DIP13	295450	microtubule binding	0.67	4	no						(Leveque et al., 2016)
7	BCC2	231070	protein kinase	0.22	2	yes						(Engelberg et al., 2021)
8	BCC5	269460	EF-hand phosphatase	-0.81	3	no						(Engelberg et al., 2021)
9	HAD2a	289910	phosphatase	-1.94	–	no						(Engelberg et al., 2016)
10	BCC8	273050	hypothetical	2.08	3	yes						(Engelberg et al., 2021)
11	BCC9	200330	hypothetical	0.26	4	no						(Engelberg et al., 2021) ^#^
12	BCC10	310220	guanylate-binding/atlastin	-1.36	3	yes						(Engelberg et al., 2021)
13	IMC5	224530	structural filament	0.31	3	no						(Anderson-White et al., 2011)
14	IMC8	224520	structural filament	1.12	3	no						(Anderson-White et al., 2011)
15	IMC9	226220	structural filament	-2.11	2	no						(Anderson-White et al., 2011)
16	IMC13	253470	structural filament	1.22	2	no						(Anderson-White et al., 2011)
17	MyoJ	257470	motor, constriction	-3.01	4	yes						(Frenal et al., 2017)
18	Cen2	250340	Ca^2+^-dependent motor filament	-4.41	4	no						(Hu et al., 2006)
19	BCC1	232780	MyoJ/Cen2 associated factor	-1.88	1/4*	yes						(Engelberg et al., 2021)
20	EB1	227650	microtubule (+)-end binding	-0.98	–	no						(Chen et al., 2015)
21	GAP80	246940	glideosome, motility	0.75	–	no						(Frenal et al., 2014)
22	IAP1	283510	glideosome, motility	1.51	1	no						(Frenal et al., 2014)
23	MyoB/C	255190	glideosome, motility, motor	2.25	2	yes						(Delbac et al., 2001; Frenal et al., 2014)
24	ELC1^§^	269442	glideosome, motility	0.11	–	no						(Nebl et al., 2011; Frenal et al., 2014)
25	DrpC	270690	constriction, organelle fission	-4.54	1	no						(Heredero-Bermejo et al., 2019; Melatti et al., 2019; Amiar et al., 2020)
26	FIKK	289050	kinase	1.42	1	yes						(Skariah et al., 2016)
27	BCC6	202550	NLI-IF phosphatase	-0.39	1	yes						(Engelberg et al., 2021)
28	MSC1a	216650	hypothetical	1.82	1	no						(Lorestani et al., 2012)
29	BCC7	311230	hypothetical (TM-domain)	0.74	1	yes						(Engelberg et al., 2021)
30	BCC11	278130	hypothetical	-0.10	3	no						(Engelberg et al., 2021)
31	BTP1	216460	TM domain	1.91	–	yes						here
32	MyoI	230980	motor, residual body	0.21	–	no						(Frenal et al., 2017)
33	CaM	249240	Ca^2+^-sensing, signaling	-5.28	–	no						(Paul et al., 2015)
34	ZFP2	212260	Zn finger	-4.50	4	no						(Semenovskaya et al., 2020)
35	GC flippase	254370	cGMP-dep. flippase; abscission	-3.56	–	no						(Bisio et al., 2019)
36	Coronin	216970	gliding	0.71	–	yes						(Salamun et al., 2014)
37	DHHC14	293730	palmitoyl transferase	-3.21	–	–						(Dogga and Frenal, 2020; Engelberg et al., 2021)
38	CDPK6	218720	Ca^2+^-dependent kinase	-0.01	3	–						(Engelberg et al., 2021)
39	kinesin	319710	motor, kinesin	-1.57	1	–						ToxoDB
40	HSP21	312600	chaperone, heat shock protein	0.55	3	–						(Engelberg et al., 2021)
41	HSP29	289600	chaperone, heat shock protein	2.28	3	–						(Engelberg et al., 2021)

‘Putative function’ is based on functional analysis and/or domains contained in the protein as annotated on ToxoDB. ‘Fitness score’ is derived from the genome wide CRISPR/Cas9 KO screen; a score of <-2.5 is a general prediction of gene essentiality (Sidik et al., 2016). ‘BCSC’ represents the BC sub-complex as defined by structural dissection of the BC by proximity biotinylation (Engelberg et al., 2021). ‘BC excl.’ indicates whether the protein exclusively localized to the BC (yes) or is also seen at other sub-cellular localizations (no). ‘Timing’ of BC association is defined as follows: in, initiation; ex, expansion; co, constriction; ma, maturation; ec, extracellular. Color coded boxes report the following: green: present at BC; white: absent from BC; grey: BC presence not determined; yellow: in the vicinity of the BC, but direct association unlikely; light green (chartreuse): tentative association with the BC.

*BCC1 co-localizes and functionally interacts with Cen2 and MyoJ in BCSC4, though by statistical analysis of BioID data shows an association with BCSC1, likely skewed by the short Cen2 sequence that was rarely detected by mass spectrometry except in its own Cen2-BioID condition (Engelberg et al., 2021).

^#^The BCC9 ortholog in P. falciparum is PfPH2 and has a role in microneme exocytosis (Ebrahimzadeh et al., 2019).

^§^ELC2 (TGGT1_305050) does not localize to the BC, but only to the IMC (i.e., the MyoA glideosome (Williams et al., 2015).

Halfway through daughter assembly, the BC thickens into an electron dense structure that appears at the basal end of the nascent cytoskeleton scaffolds. At this point the cytoskeletons are being assembled within the cytoplasm of the mother cell (**Figures 2A, C**). Here, the BC constricts, mediated by Myosin J (MyoJ) and Centrin2 (Cen2) (Hu et al., 2006; Frenal et al., 2017), and likely works together with a recently discovered component, BCC1 (Engelberg et al., 2021). At the same time, additional cytoskeleton proteins (Anderson-White et al., 2011) as well as phosphatases and kinases are recruited. MyoC is deposited at the BC at this point as well, although its key function occurs much later in motility of the mature parasite (Delbac et al., 2001; Frenal et al., 2017; Tosetti et al., 2019). Over 20 proteins are present at the BC at the time of its constriction, which resolve into four different BC sub complexes (BCSC) based on statistical analysis of proximity biotinylation data [BCSC1-4; **Table 1**; (Engelberg et al., 2021)]. This mirrors the several substructures discernable by electron microscopy (**Figures 2A, C**). Loss of either MyoJ or Cen2 from the BC results in incomplete BC constriction but has only a modest effect on parasite fitness *in vitro*, suggesting this step is not strictly essential (Frenal et al., 2017). This observation fits with the modest effect of actin depolymerizers on daughter cell formation (Shaw et al., 2000) and that the deletion of the actin gene does not prevent the completion of cell division (Periz et al., 2017).

While the BC is constricting, the extension of the subpellicular microtubules stops and their (+) ends dissociate from the BC when they are at about 2/3 of the final length of the parasite. At this point the microtubule end binding protein EB1 is briefly visible at the BC, although it is not clear if it directly associates with the complex [**Table 1** (Chen et al., 2015)]. This event most likely coincides with DIP13 dissociation from the BC (Leveque et al., 2016), and while the mechanism is not well understood, its timing overlaps with completion of karyokinesis, an event in which EB1 has a dynamic role as well (Chen et al., 2015). The next BC transition occurs when the plasma membrane is deposited on the IMC: an electron-dense connection from the BC folding around the end of the IMC to the plasma membrane is now formed (**Figures 2A, D**) (Anderson-White et al., 2011). Dynamically, this is when the BC reaches its most constricted state. At this point BCC4 releases from the BC (Engelberg et al., 2021) (**Table 1**). Functionally, this BC constellation is associated with several different events late in the cell division process: completion of plastid and mitochondrial division and partitioning, depositing of mother remnants into the residual body and their subsequent recycling (i.e., re-uptake in the daughters), and finally the maintenance of a cytoplasmic bridge between the daughter parasites upon completion of cell division (Frenal et al., 2017; Periz et al., 2017; Hunt et al., 2019; O’Shaughnessy et al., 2021).

Following complete daughter separation, the BC connection from the inside of the IMC to the plasma membrane is lost and the structure transforms into the much more electrolucent posterior cup, which has a small pore at the very basal tip (Mann and Beckers, 2001) (**Figure 2E**). This complete separation requires the activity of an unique guanylate cyclase (GC) flippase protein present at the BC (Bisio et al., 2019).

**Figure 3:**
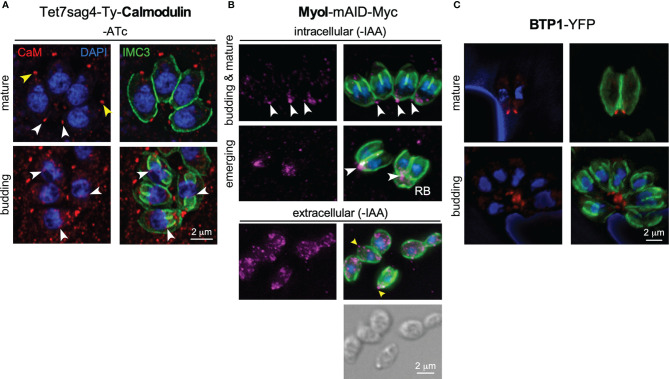
Additional players at the basal complex. **(A)** Endogenously tagged Calmodulin localizes to the BC in mature parasites and the daughter buds upon constriction (white arrowheads), next to a more prominent localization at the apical end (yellow arrowheads). IMC3 marks the cortical cytoskeleton of mother and daughter parasites; DAPI marks the DNA. CaM depletion and phenotype upon ATc treatment shown in **Figures S1A–C**. **(B)** Endogenously tagged MyoI displays a spotty pattern reminiscent of vesicles. MyoI accumulates in and/or around the BC in mature parasites and in the (forming) residual body (RB), as marked by white arrowheads. In extracellular parasites the signal is more diffuse throughout the cytoplasm but a prominent signal around the BC is observed in many parasites as marked by yellow arrowheads. MyoI depletion upon IAA treatment shown in **Figure S1D**. **(C)** Exogenously expressed BTP1 tagged with YFP (false colored red) under its native promoter localizes to the BC in the mature cytoskeleton only. IMC3 marks the cortical cytoskeleton of mother.

Finally, upon egress from the host cell into the extracellular environment, the parasites become motile. MyoB/C contributes to gliding motility and subsequent invasion of the next host cell (Frenal et al., 2014). In motile parasites, the F-actin stabilizing protein Coronin is recruited to the BC in response to changes in intracellular Ca^2+^ (Salamun et al., 2014), which might be mediated by calmodulin at the BC ((Paul et al., 2015); **Figure 3A**). Coronin has also been tangentially associated with microneme secretion, likely due to its putative role in endocytosis occurring at the basal end to offset membrane surplus by microneme secretion at the apical end of the cell (Gras et al., 2019).

**Figure 4:**
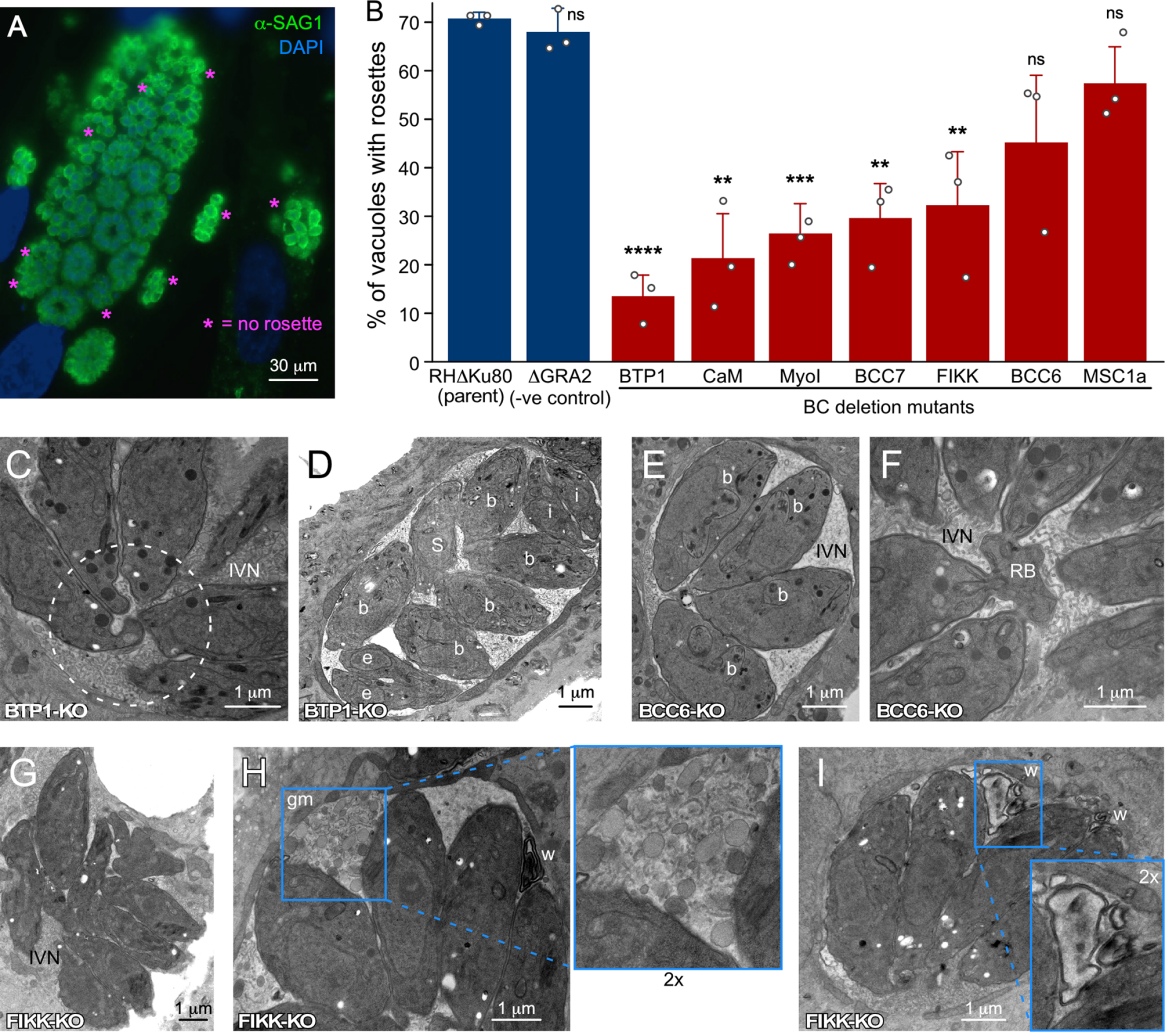
Ultrastructural assessment of the PVM, IVN and intravacuolar organization of parasites wherein genes encoding BC proteins recruited upon completion of division have been knocked out. **(A)** IFA example of rosette quantification assay. Shown is the parent line control (RHδKu80). **(B)** Incidence of parasites organized in rosettes. Only vacuoles with >16 parasites per vacuole were counted and at least 100 vacuoles per biological repetition were counted. Bars represent the average of three biological repetitions, error bars report standard deviation. Student’s *t*-test relative to RH∆Ku80: * < 0.05; ** < 0.01; *** < 0.001; **** < 0.0001; ns, not significant. **(C, D)** BTP1-KO parasites are not connected by a cytoplasmic bridge or to the residual body (C; dotted circle) and divide asynchronously D; b, budding; e, emerging; S, S-phase; i, interphase/mature), but display normal IVN formation. **(E, F)** BCC6-KO parasites show no defects at all and are shown as reference for the wild type manifestation of the IVN, synchrony in cell division and connections of the basal ends to the residual body. **(G–I)** FIKK-KO parasites are not well-organized in the PV **(G)**, display abundant ‘gray matter’ (gm) inside the vacuole of unknown origin or composition **(I)**, and frequently display electron-dense membrane whirls (w) inside the vacuole **(H, I)**. Where relevant, parasite development stages are marked as follows: b, internal budding; e, emerging daughters; s, S-phase; i, interphase. IVN, intravacuolar network; RB, residual body; gm, gray matter; w, membrane whirls inside the PV.

Upon conclusion of the host cell invasion process, the parasite twists to seal off the vacuole (Pavlou et al., 2018), resulting in a twisted appearance of the basal end (**Figure 2A**, invaginated). It is not clear whether the BC has an active role in this process or whether the distorted BC appearance might be a physical result of the contortion accompanying twisting. Shortly after completion of invasion, the parasite starts to assemble the IVN at the basal end, a process where an active role of BC is yet to be demonstrated (**Figure 2A**) (Sibley et al., 1995). It is not clear whether the twisted and invaginated appearances of the BC are (functionally) related to each other either, but such appearances are sometimes seen after multiple rounds of division as well (e.g., **Figure 4C**). Thus, current knowledge on the BC during assembly, cell division, and cell-cell communication is quite detailed, but our understanding of BC manifestation and function during invasion and the assembly and/or function of the IVN is still very limited.

## Cell Division

The distinct ultrastructural presentation and key composition changes of the BC during cell division are discussed in the previous section. The prominent role of the BC during cell division is to function as the contractile ring and to separate the daughters. The mechanism was a puzzle for a long time since depletion of the obvious MyoJ motor complex disabled BC constriction but only caused a very minor growth phenotype (Frenal et al., 2017), while depletion of BC scaffolding protein MORN1 is lethal and results in conjoined daughters unable to separate from each other (Heaslip et al., 2010; Lorestani et al., 2010). This paradox suggests the presence of an additional contractile event preceding the recruitment of the MyoJ/Cen2/BCC1 complex at the midpoint of budding. We recently described a stretchy rubber band model of the BC, where it is the stability of an expandable BC ring with MORN1 and BCC4 as key players, that is the most critical function of the BC during cell division. In absence of either component, the BC is initially assembled but at the midpoint the complex suddenly breaks into pieces, which results in fraying of the extending subpellicular microtubules (Engelberg et al., 2021). This effect is best described as opening an umbrella and results in daughter buds that have very wide basal diameters, which prevents their separation (these conjoined daughters have been dubbed the ‘double-headed’ or ‘multi-headed’ phenotype). Resolving this conundrum was a major breakthrough in understanding the key role of the BC during cell division.

There are several additional phenomena during cell division with crucial roles of the BC. BC formation is first visible as MORN1 and BCC4, which are assembled on a five-fold symmetrical structure. This foundational structure comprises proteins that form the basis for the three distinct major components of the cytoskeleton scaffolds (microtubules, alveolar vesicles, epiplastins/alveolins) (Engelberg et al., 2021). IMC32 and F-BOX ubiquitin ligase (FBXO1) are required for IMC membrane skeleton formation (Baptista et al., 2019; Torres et al., 2021), whereas AC9 is critical in conoid and subpellicular microtubule formation (O’Shaughnessy et al., 2020; Tosetti et al., 2020). In addition, two proteins, BCC0 and BCC3, also exhibit this distinct 5-fold symmetry and were specifically detected in proximity biotinylation experiments using BC components as bait (Engelberg et al., 2021). Further catering to this assessment is that BCC0 has been posited as a potential ortholog of the radial spoke protein 5 (RSP5) in *Chlamydomonas reinhardtii*, which binds to MORN domain proteins, although the statistical support for this homology is moderate at best (Portman et al., 2014; Portman and Slapeta, 2014). Furthermore, BCC3 is deposited in the early BC from which it transitions into the sutures between the alveolar membrane sheets that make up the IMC. This suggests a direct connection between the five-fold symmetry of the alveolar structure and the BC as well as extending the daughter buds. An additional player in IMC formation is palmitoyl acyltransferase DHHC14, which has been functionally associated with IMC formation (Dogga and Frenal, 2020). Moreover, DHHC14 is prominently detected in the BC by proximity biotinylation (Engelberg et al., 2021) and together these insights support a model wherein the BC serves as the docking site to add new cytoskeletal components by palmitoylating proteins deposited into the alveolar vesicles. Furthermore, addition of Golgi derived vesicles at the basal end of daughter buds is mediated by Rab11b GTPase (Agop-Nersesian et al., 2010), which ties the BC into this process. These observations all align with the apical to basal direction of the daughter assembly and put the BC at the place of daughter bud extension. This model comes with an exception: the apical cap section of the alveolar membrane skeleton appears to bud in the opposite, apical direction away from the five-fold symmetrical foundational structure (Engelberg et al., 2021).

Furthermore, induced defects in apicoplast lipid metabolism, either pharmacologically by ciprofloxacin [acting indirectly by acting on DNA gyrase; (Martins-Duarte et al., 2015)] or triclosan [acting directly by inhibiting FASII (Martins-Duarte et al., 2016)], or genetically by disrupting an apicoplast acyl carrier protein (ACP) (Martins-Duarte et al., 2016) or acetyltransferase 2 (ATS2) (Amiar et al., 2020), result in a cell division stall at the late stage when the mother’s plasma membrane is added and daughter buds start to emerge. Incomplete BC constriction is also observed here which therefore seems to be tied, directly or indirectly, to the addition of IMC and/or plasma membrane to the emerging daughters. Interestingly, in the ATS2 depleted mutants, DrpC localization to the BC of dividing daughters is defective, which due to its putative membrane constrictive properties, is suggested to be the cause of the lack of constriction [(Amiar et al., 2020) and DrpC docking on the BC was shown to be dependent on the presence of phosphatidic acid]. Reciprocally, direct depletion of DrpC does results in two different phenotypes: 1) defective delivery of vesicles to the growing IMC (Heredero-Bermejo et al., 2019; Amiar et al., 2020) and 2) incomplete mitochondrion division [(Melatti et al., 2019) further discussed below]. Overall, these phenotypes suggest a model wherein fatty acid production in the apicoplast ensures the availability of phosphatidic acid at the BC of budding daughters. This secures the recruitment of DrpC, which in turn, is needed to add more membrane to the IMC and/or plasma membrane. Finally, another pharmacological agent inducing a similar phenotypic block in plasma membrane addition is itraconazole, which at least in fungi inhibits sterol metabolism and thus, most likely affects membrane biogenesis in *T. gondii* (Martins-Duarte Edos et al., 2008). Taken together, vesicular membrane docking at the BC is required to complete cell division.

## Organelle Division

Firstly, completion of apicoplast division is dependent upon MORN1 (Lorestani et al., 2010). During apicoplast division, the extended organelle is anchored in the daughter buds by association of the ends to the centrosomes while the undivided organelle is seen extending to the basal ends of the daughter cytoskeleton buds with a sharp bend where the basal complexes meet (Vaishnava et al., 2005; Gubbels et al., 2006). Furthermore, the dynamin related protein A (DrpA) is enriched at the pinching point of the apicoplast where the BCs of the daughters come together (van Dooren et al., 2009). However, whether DrpA is specifically recruited to the BC or whether DrpA localizes to the physically most strained plastid region has not been established. In chronological order, apicoplast division completes at the onset of BC constriction, i.e., coinciding with the assembly of the electron dense bulb containing the IMC proteins, MyoJ and Cen2 (**Figure 2C**).

Secondly, the single copy mitochondrion of *T. gondii* is the last organelle entering the daughter buds around the time when the plasma membrane deposition is completed and the residual body is formed (**Figure 1**) (Nishi et al., 2008). The mitochondria are anchored to the daughter bud IMC through lasso-mitochondria factor 1 (LMF1) (Jacobs et al., 2020), whereas their extended membrane structure is directed into the daughter buds in a DrpC dependent fashion (Melatti et al., 2019). This phenomenon again might be mediated by DrpC-dependent vesicular transport (Heredero-Bermejo et al., 2019; Amiar et al., 2020). In conclusion, the BC has been associated with completion of apicoplast segmentation and partitioning of the mitochondrion but the exact mechanism in both cases is not well resolved.

## Residual Body and Cytoplasmic Bridge

In the late stages of division, when the daughter cells emerge from the mother, remnants from the mother are deposited in the residual body. The residual body sits at the basal end of the daughters and quickly disappears following completing of division (e.g., **Figure 2C**). Thus, the residual body is a recycling bin that is quickly emptied. There are two models of what happens with the residual body and its digested content, which may both be true: 1) the residual body is slowly degraded inside the vacuole and is assimilated into the surrounding IVN tubules, and 2) the digested contents are resorbed into the daughters that are still connected to the residual body. The former model could apply if the residual body gets severed from the daughter cells. However, it is frequently observed that after the residual body is emptied, the connection between daughter parasites, known as the cytoplasmic bridge, is maintained. This ‘cytoplasmic bridge’ facilitates cell-cell communication between parasites and results in synchronized cell division cycles within the vacuole (Frenal et al., 2017; Periz et al., 2017; Hunt et al., 2019). Upon several sequential division rounds, the cytoplasmic bridge connects multiple parasites in the vacuole and is also responsible to maintain rosette organization of parasites (Muniz-Hernandez et al., 2011). Since the connection originates at the basal end, the BC is in a key position to function in the residual body and the cytoplasmic bridge. MyoI is an essential player in establishing and/or maintaining the cytoplasmic bridge (**Figure 2C**), with a supportive role assigned to MyoJ (Periz et al., 2017). Why the cytoplasmic bridge is maintained is not known as depletion of MyoI and does not cause a change in parasite fitness (Periz et al., 2017). Although presence of the bridge ensures synchronized division, an obvious need to maintain synchronized cell cycles is not evident. In fact, the bridge is lost in activated macrophages (possibly due to external force acting on the vacuole) and during bradyzoite differentiation, facilitating the asynchronized, slow division in the tissue cysts (Frenal et al., 2017).

We reasoned that besides MyoI, proteins recruited to BC upon completion of cell division might contribute to stabilizing the cytoplasmic bridge (**Table 1**). This list is comprised of the FIKK kinase (Skariah et al., 2016), CaM [**Figure 3A**; (Paul et al., 2015)], and MSC1a, a protein of unknown function (Lorestani et al., 2012), and two new proteins (BCC6, a phosphatase and BCC7, a hypothetical protein), which were recently added to this list (Engelberg et al., 2021). Furthermore, here we mapped one additional protein: the *T. gondii* ortholog of *P. falciparum* transmembrane protein 1 (PfBTP1), which was linked to acquisition of the plasma membrane by the emerging merozoites (Kono et al., 2016). We cloned the genomic sequence of TgBTP1 including the upstream promoter region in-frame with a YFP reporter gene and observed an exclusive localization to the mature BC and to some extent, in the residual body (**Figure 3C**). Collectively, six out of seven proteins associated with the BC upon maturation have fitness scores suggesting they are dispensable for the lytic cycle (Sidik et al., 2016), whereas fitness-conferring CaM is non-exclusively localizing to the BC and likely has other functions that are essential (**Figure 3A**; **S1**) (Paul et al., 2015). Indeed, we were able to generate complete knock-outs for all six genes (**Figure S2**) and generated a conditional knock-down line for CaM using the Tet-off system (Meissner et al., 2002). None of the mutant displayed any significant reduction in plaque forming capacity (**Figure S3**). We first analyzed whether these mutants had a cytoplasmic bridge phenotype by assessing the incidence of parasite organization in rosettes within the vacuole (**Figures 4A, B**). In comparison to wild type parasites where 70% of the vacuoles display a rosette organization, the mutants displayed more diversity; BTP1 and CaM knockout parasites exhibited rosettes in only 15-20% of vacuoles, while for MyoI, BCC7, and FIKK knockouts rosettes were observed in 35-45% of the vacuoles. For BCC6 and MSC1a-depleted parasites, we found no significant difference in amount compared to wild type parasites. Thus, these observations strongly suggest that most mature BC proteins support, in one way or another, the formation and or maintenance of the cytoplasmic bridge.

Subsequently, we dissected representative KO mutant parasite strains at the ultrastructural level (**Figures 4C–I**). This confirmed that BTP1-KO parasites lost the cytoplasmic bridge (**Figure 4C**), and as a result displayed vacuoles harboring non-synchronously dividing parasites (**Figure 4D**). Conversely, BCC6-KO readily showed synchronously dividing parasites (**Figure 4E**) and connections to the residual body (**Figure 4F**), consistent with the high incidence of rosettes (**Figure 4B**). We observed random organization of FIKK-KO parasites in the vacuole as reflected in the rosette assay (**Figure 4G**), and, in addition, the vacuoles displayed abnormalities in IVN membrane organization such as undefined globules of gray matter (**Figure 4H**) and membrane whirls (**Figure 4I**). This suggests that the proteins associating with the mature BC could also impact IVN formation and/or function.

In conclusion, the most prevalent phenotype seen in parasites depleted of proteins associating with the mature BC is the loss of the cytoplasmic bridge and associated secondary phenotypes, such as inability to form rosettes or divide in synchrony. Since we were unable to determine any fitness consequences for the mutant parasites under our experimental conditions, the functional relevance of these numerous BC proteins is currently unclear. It is of note that depletion of LMF1, which anchors the mitochondria in the IMC, also interferes with rosette formation, which might suggest that the cytoplasmic bridge is maintained to accommodate the mitochondrion (Jacobs et al., 2020).

## Motility

MyoC resides at the site of the BC and is already seen at the BC halfway during the division process [**Table 1**, MyoB is splice variant of the same gene (Delbac et al., 2001)]. However, MyoC does not function in cell division but is involved in gliding motility and host cell invasion (Frenal et al., 2014). It can replace the function of another class XIV myosin, MyoA, which however alters the tachyzoite’s invasion process and efficiency (Bichet et al., 2016). MyoC is restricted to the posterior end of the cell by IMC-associated protein 1 (IAP1) and is in complex with glideosome associated protein 80 (GAP80), which forms a bridge between the BC and plasma membrane (Frenal et al., 2014), and Ca^2+^-binding essential light chain 1 (ELC1), but not ELC2 (Nebl et al., 2011; Frenal et al., 2014). Thus, the MyoC complex does not reside on the cytoplasmic side of the IMC but on the outside facing the plasma membrane. The reason for a specialized MyoC at the BC is not known, as its depletion has no impact on viability and MyoC orthologs are not widely conserved within the Apicomplexa (Frenal et al., 2014). It is possible that MyoC is essential in other developmental stages or specialized motility modes, which have not yet been probed.

Another distinct role for the BC in motility is the presence of actin filament binding coronin. Coronin translocation from the cytoplasm to the BC only occurs in extracellular parasites, is Ca^2+^-dependent, and is correlated, likely in a co-dependent fashion, with microneme protein discharge (Salamun et al., 2014). Recently, motility was associated with the need for endocytic activity in extracellular parasites, which most logically would occur at the posterior end of the parasites, possibly the BC (Gras et al., 2019). Thus, coronin-mediated endocytic activity at the BC could be required for efficient motility. Interestingly, *Plasmodium* coronin has also been associated with parasite endocytosis (Bisio et al., 2019) and supports this assignment, although this assignment was made in a different context (hemoglobin uptake rather than motility). Furthermore, MyoI has been shown to be critical for extracellular survival (Primo et al., 2021) and might be associated with this process as well. Although we occasionally observe MyoI at or around the BC of extracellular parasites, MyoI foci are widely distributed throughout the cytoplasm, which therefore, only partly supports this hypothesis and needs further attention in the future (**Figure 3B**) (Frenal et al., 2017). An additional, more puzzling observation is found in the TgBCC9 ortholog in *P. falciparum*, named PfPH2, which was associated with a role in microneme exocytosis and localizes to the apical end (Ebrahimzadeh et al., 2019; Mukherjee et al., 2022). As evident from the data assembled here, endocytosis of extracellular parasites might play out at the basal end rather than at the apical end and could be mechanistically related to the microneme secretion defect observed upon coronin depletion in *T. gondii* (Salamun et al., 2014). However, as discussed in detail below, the low conservation of BC proteins between *T. gondii* and *P. falciparum*, in general, might be reflected in a truly distinct function of this seemingly orthologous gene in each organism. Additional work is needed to conclusively tie these processes together at the BC of the parasite.

## PVM and IVN Formation

In the last steps of the host cell invasion process, the parasite has to seal off the host cell’s plasma membrane, as well as seal the forming parasitophorous vacuolar membrane (PVM). Recent work has shown that a twisting motion by the parasite inside the vacuole mechanically induces host cell membrane fission and PVM sealing to complete the invasion within a protective vacuole (Pavlou et al., 2018). Since this sealing occurs at the posterior end of the parasites, which is the last part of the parasite to enter the cell and PVM, the posterior end of the parasites often appears twisted in newly invaded parasites (**Figures 1**, **2A**). In addition, contorted parasites are sometimes also seen among parasites within larger vacuoles [e.g., the far left parasite in **Figure 4C** or as reported in (Venugopal et al., 2017)]. Although the BC appears to be at the center of the action here, there are currently no experimental data to support a direct and/or active role.

Another event that unfolds at the posterior end of the parasite, specifically at the site of the BC, is a 10-15 min post completion of invasion which is the formation of the intravacuolar tubulovesicular network (IVN, tubulo-vesicular network, or TVN) (**Figures 1**, **2A**). The IVN is composed of a membranous interface derived from multi-lamellar vesicles secreted by the parasite (Dubremetz et al., 1993; Sibley et al., 1995) and lipids scavenged from the host (Caffaro and Boothroyd, 2011). The IVN is stabilized by various dense granule proteins (GRA2 and GRA6) secreted by the parasite (Labruyere et al., 1999; Mercier et al., 2002; Travier et al., 2008). Deletion of GRA2 and/or GRA6 results in loss of the IVN (Mercier et al., 2002) and subsequent poor acute virulence in mice (Mercier et al., 1998; Alaganan et al., 2014) [likely due to impairment of the CD8 response (Lopez et al., 2015)], and markedly reduced chronic infection (Fox et al., 2011). However, “how” the IVN is established and maintained is far from resolved. Notably, the debate regarding the site of dense granule secretion in *Toxoplasma* is not settled either. For example, different dense granule populations with different contents exist and not all may be secreted at the same time, site, or by the same mechanism (Mercier and Cesbron-Delauw, 2015). Although there is evidence that the dense granules are secreted somewhere at the apical end of the parasite, possibly through the apical annuli (Dubremetz et al., 1993; Engelberg et al., 2020), their secretion has also been observed from a specialized invagination at the posterior end of the parasite where the BC resides (Sibley et al., 1995). It was initially reported that GRA2 is released from the posterior end of the parasite as several multilamellar vesicles emanating from the invaginated BC area, however, another study reports that GRA2 is secreted apically and then recruited to the posterior end (Mercier et al., 1998). In either scenario GRA2 multilamellar vesicles are deposited in the vacuole at the site of the invaginated BC, which are subsequently assembled into tubulated structures making up the mature IVN (Sibley et al., 1995) (**Figures 1**, **2A**). We note that a block of all dense granule secretion would abrogate the secretion of GRA17 and GRA23, which form a pore in the vacuolar membrane and their deletion results in distinctly, osmotically swollen vacuoles (Gold et al., 2015). For the BC mutants tested, we have not observed such swollen vacuoles and as such our data, so far, do not support a role of the BC in dense granule secretion.

During IVN formation the BC has an indented appearance and a direct role for the BC is quite likely, at least for structural support but direct evidence is absent. We reasoned that BC proteins recruited to the BC following completion of division could potentially function in this process. As mentioned, neither BTP1 nor BCC6 mutants displayed any defects (**Figures 4C–F**). However, FIKK-KO parasites show IVN abnormalities (**Figures 4G–I**): 1) abundant ‘gray matter’ inside the vacuole of unknown origin or composition, which is sometimes also seen in wild type parasites; 2) presence of electron-dense membrane whirls inside the vacuole, also of unknown origin. In summary, none of the three tested mutants affected IVN formation but loss of FIKK leads to abnormal structures, which at the very least, does associate the BC with processes within the vacuole.

## Nutrient Acquisition

**Figure 5:**
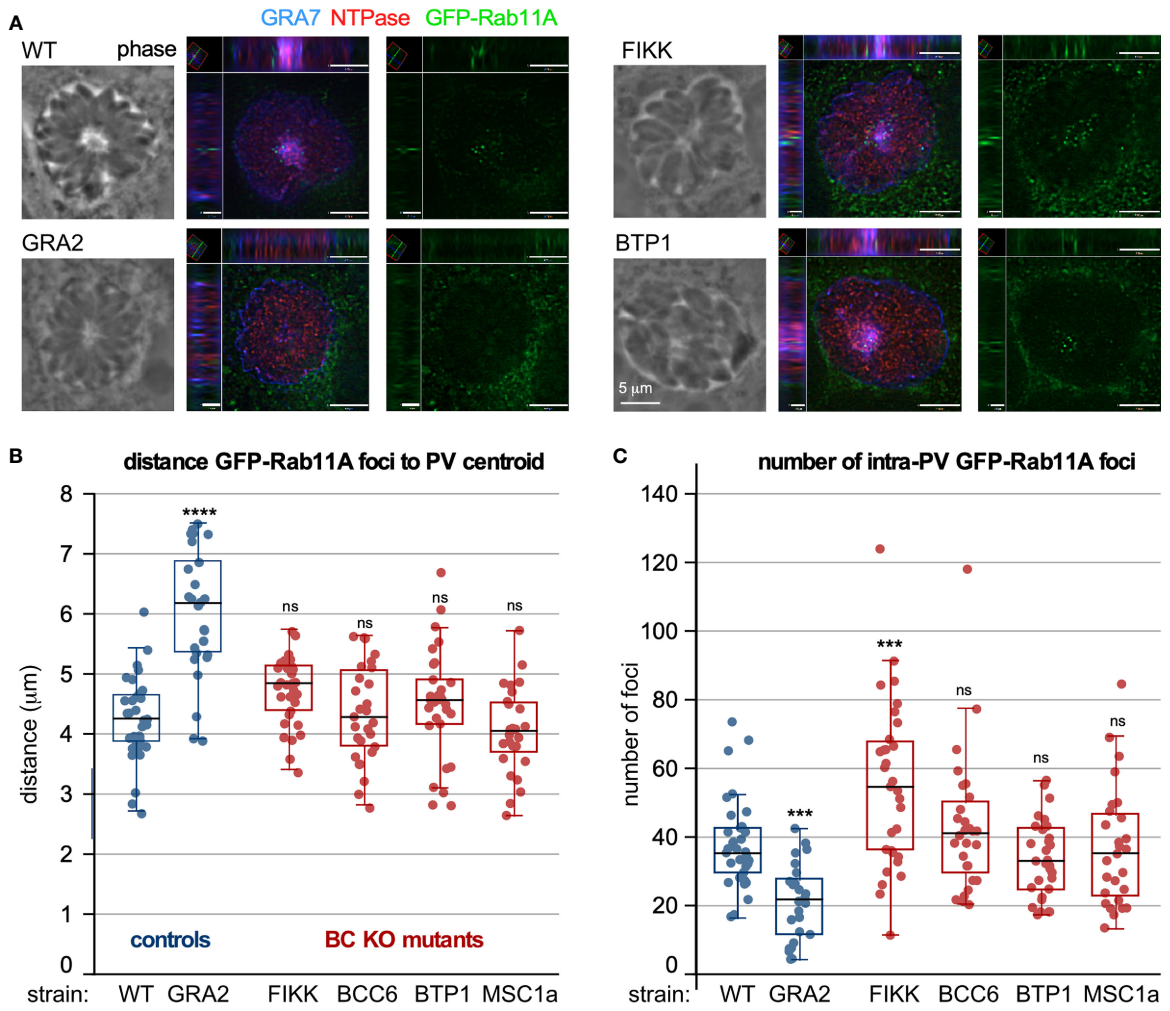
The role of BC proteins recruited upon completion of division in IVN function. **(A–C)** RH strain parasites with BC protein gene knock-outs and controls were inoculated in host cells expressing GFP-Rab11A, which accumulate in the center of the vacuole where the basal ends are clustered (Romano et al., 2017). GRA2-KO parasites do not form an IVN and were used as negative control. **(A)** shows two examples of XYZ projections on which measurements were performed. Shown are wild type and mutant parasite infected samples stained additionally with GRA7 to highlight the parasitophorous vacuole membrane and IVN and NTPase to mark the parasitophorous vacuole lumen. **(B)** Measurements of distance of the GFP-Rab11A foci inside the vacuole to the centroid using Volocity software. **(C)** The total number of foci per vacuole was counted. For B and C, the experiment was performed once and at least 26 vacuoles were counted; box plots depict average ±SD with whiskers representing the upper and lower values excluding outliers; the black line inside the box is the median. ANOVA + Tukey’s HSD relative to RH∆Ku80: *** < 0.001; **** < 0.0001; ns, not significant. All source data, including additionally acquired metrics that did not reveal statistical differences are provided in **Table S4**. **(D)** Prugniaud strain BC protein knock-out and control parasites were i.p. injected in female C57/BL6 mice (group 1 and 3: 500 tachyzoites; group 2 and 4: 2500 tachyzoites). Brains were harvested 3-4 weeks post infection and cysts purified from two pooled mice brains and enumerated following DBA staining of the cyst wall. n.d., not done.

Intracellularly residing parasites can acquire nutrients from the host cell in several ways (Coppens et al., 2006; Romano et al., 2013; Dou et al., 2014; Gold et al., 2015; Romano et al., 2017; McGovern et al., 2018). Germane to a role of the BC in this process is the observation that host derived vesicles accumulate in the parasitophorous vacuole (PV). Specifically, host GFP-Rab11A decorated vesicles accumulate in the center of the PV in the middle of the rosette at the basal ends of the replicating parasites (Romano et al., 2017), and strongly suggest the participation of the BC in host vesicle remodeling and/or lipid uptake (Romano et al., 2017) (**Figure 5A**). We utilized our set of parasite BC gene KO strains to determine any changes of host GFP-Rab11A accumulation patterns. As a positive control we generated a GRA2-KO mutant, which in a different genetic background strain was previously shown to abrogate IVN formation and to be critical to this process (Romano et al., 2017). GRA2 depletion indeed resulted in an increased distance between GFP-Rab11A vesicles and the vacuole centroid, with GFP-Rab11A vesicles localized around the PV membrane (**Figure 5B**) and a decrease of the total number of vesicles observed per vacuole (**Figure 5C**). However, none of the BC mutants show significant changes in foci distance from the vacuole center and only the FIKK-depleted mutant showed an increase in number of vesicles per parasite (**Figures 5B, C**). Thus, FIKK depletion leads to an accumulation of vesicles in the PV, which is most likely the result of the aberrant membrane whirls and gray matter forming in the vacuole upon FIKK depletion.

We recently discovered a role for MyoI in extracellular parasites, as increased MyoI expression was associated with prolonged extracellular survival capacity (Primo et al., 2021). Moreover, extracellular survival capacity was functionally mapped to the availability of fatty acids. As such, a plausible model is that lipid uptake by extracellular parasites is mediated by MyoI at the BC (**Figure 3B**). Furthermore, uptake of extracellular material would be consistent with endocytosis during gliding but whether the mechanism is the same remains to be directly tested.

## Tissue Cyst Formation

All insights discussed so far pertain to the tachyzoite life stage of *Toxoplasma*. However, insights have been emerging for a role of the BC in chronic infection of mice, which is established by differentiation of tachyzoites into bradyzoites. Bradyzoites are not very proliferative or metabolically active and reside within tissue cysts contained by a proteoglycan cyst wall (Tu et al., 2018; Cerutti et al., 2020). A putative role for the BC in bradyzoites was revealed by the identification of the FIKK mutant that resulted in sharply reduced chronic infection in a mouse model (Dr. Dana Mordue, personal communication). Furthermore, cyst formation is dependent on the IVN as disruption of key GRA proteins (e.g., GRA2) disrupts IVN assembly and severely impacts the ability to establish chronic infection (Fox et al., 2011). It is important to note that the bradyzoite IVN is different from the discrete tubules seen in tachyzoites: the vacuolar space in bradyzoites is a matrix of differently sized vesicles and tubules organized in a network that connects the bradyzoites to each other and to the cyst wall. Moreover, this matrix is involved in the transport of materials from the bradyzoites to the cyst wall (Ferguson and Hutchison, 1987; Lemgruber et al., 2011; Tu et al., 2018). The BC could therefore have a putative role in cyst maintenance and/or function of the matrix. We set out to test whether proteins recruited to the mature BC affect the ability to establish and/or retain brain cysts in mice. Since the already generated KO mutants were in the Type 1 genotype RH strain that is hypervirulent in mice and does not permit the formation of cysts, we generated direct KOs of FIKK, BCC6, BTP1, and MSC1a genes, while GRA2 was used as a positive control in the genotype II Prugniaud (Pru) parasite strain. As expected, the cyst load was severely reduced in both GRA2-KO and FIKK-KO infected mice (**Figure S4**). However, none of the other mutants displayed a strong reduction in cyst loads. Thus, we conclude that amongst the proteins recruited to the mature BC, only FIKK impacts chronic infection, and hence, the other tested BC proteins are not involved in cyst formation in the bradyzoite stage.

## Outlook

In summary, the BC is a multifunctional structure with several validated and a series of putative functions. Regarding cell division, the BC is the site where building blocks are added to the cytoskeleton scaffold, the BC has a two-step mechanism (stretch and constriction) as contractile ring, and the BC is key in organelle division. Furthermore, the BC has roles in ‘import’, i.e., the acquisition of host-derived vesicles, possibly the acquisition of extracellular lipids and the endocytosis of microneme proteins to facilitate motility. In parallel, the BC is also tied to motility through the presence of MyoC. Furthermore, the BC acts on IVN assembly in a poorly understood fashion, that may directly or indirectly contribute to the establishment and/or maintenance of chronic tissue cysts.

The most remarkable aspect regarding the BC as contractile ring in cell division is that a motor protein is strictly obsolete. Notably, other apicomplexans, like *Plasmodium falciparum*,seem to have done away with it altogether (Rudlaff et al., 2019; Morano and Dvorin, 2021). Indeed, many protozoa complete cell division without an actinomyosin ring (Balasubramanian et al., 2012; Hammarton, 2019). Since the apicomplexan cell division process shares many features with the assembly of cilia or flagella (Portman and Slapeta, 2014), we hypothesize that the actinomyosin ring together with Cen2 has evolutionary shared ancestry with “transition zone” at the base of motile cilia, sensory cilia, and the connecting cilium in photoreceptor cells (Trojan et al., 2008). Particularly in photoreceptor cells, centrin/G-protein complexes organize signaling in retinal photoreceptor cells, next to a function as a barrier and facilitate transport of vesicles (Trojan et al., 2008). In Apicomplexa this function may still be intact (e.g., in recycling material from the residual body and/or mitochondrion partitioning) but appears to have been repurposed for additional roles in cell division.

Studies on the BC in *P. falciparum* have identified a set of proteins that is largely not conserved in *T. gondii* (Rudlaff et al., 2019). A notable player is PfCINCH, a dynamin-like protein with an essential role in *P. falciparum* BC constriction. In *T. gondii* DrpC is present in the BC but is involved in transport rather than BC constriction (Heredero-Bermejo et al., 2019; Melatti et al., 2019). A putative ortholog of PfCINCH is BCC10 (Engelberg et al., 2021) as it displays homology with atlastin; in vertebrates atlastin is a dynamin-like GTPase required for homotypic fusion of endoplasmic membrane tubules (Betancourt-Solis et al., 2018). However, we did not identify an orthologous protein of BCC10 in *P. falciparum* (Engelberg et al., 2021), whereas the mild fitness score indicates BCC10 is not essential, suggesting it is not a functional PfCINCH ortholog. These examples are part of a bigger picture: only very few of the BC components mapped in *T. gondii* and *P. falciparum* are conserved, and if they are conserved, their functions diverged [e.g., MORN1 is widely conserved across division modes (Ferguson et al., 2008), but unlike TgMORN1, PfMORN1 is not essential (Moran and Dvorin, 2021)]. Another example is the single FIKK gene in *T. gondii*, which contrasts sharply with the expanded FIKK family in *P. falciparum* with functions in non-cell cycle related processes outside the parasite boundaries (Schneider and Mercereau-Puijalon, 2005; Lim et al., 2012). This is puzzling and possible scenarios are that these proteins evolve very fast. This is supported by the many coiled-coil regions as the only discernable feature in many of the BCC proteins. At the same time, these regions might be reflective of the fundamentally different budding strategy in *T. gondii* vs. *P. falciparum*: internal budding in the cytoplasm of the mother vs. external budding outward at the plasma membrane of the mother cell, respectively (Gubbels et al., 2020). Further work is needed to answer these questions.

Besides the architectural and functional divergence of the BC within the Apicomplexa, there are several additional open questions regarding specific *T. gondii* BC proteins and their functions. The first one is the puzzling set of BC proteins only acquired upon completion of cell division. We show that several function in maintenance of the cytoplasmic bridge but this bridge is not essential, at least not in tachyzoites. Although we hypothesized these might be involved in import, genetic dissection of the components so far did not identify strong support for this hypothesis. The pursuit of the alternative hypothesis that these proteins function in IVN assembly did not find much support, besides a minor role for FIKK. However, we did confirm the previous observation that FIKK is key to establish chronic infections in mice (Dr. Dana Mordue, personal communication) but none of the other BC proteins in this group contributed to this process.

The disruption of FIKK does not abolish IVN formation, yet sharply reduces the cyst loads. Notably, a similar phenotype was reported for GRA12 depleted parasites, which display an apparently intact IVN but exhibit a delay in the accumulation of the CST1 major cyst wall protein at the cyst periphery (Fox et al., 2019). Involvement of the IVN in stage differentiation is unlikely to be related to nutrient acquisition since differentiation in response to nutrient stress is well documented (Skariah et al., 2010; Sullivan and Jeffers, 2012). Since the IVN as seen in tachyzoites looks very different in bradyzoites, as a conglomerate of vesicles and tubules, its function might be different (Ferguson and Hutchison, 1987; Lemgruber et al., 2011; Tu et al., 2018). A putative role for the BC in remodeling and/or maintaining the ‘IVN’ in cysts might be dependent on the BC but beyond FIKK, we found no further experimental support for this scenario. As such, the critical function of most BC proteins associated with mature parasites remains largely unknown.

Another dimension of the BC that is still shrouded in many questions is how its elaborate architecture relates to the variety of functions ascribed to the BC. As shown in **Figure 2** and **Table 1**, BC structure and composition changes significantly throughout tachyzoite development. An established structure-function is that the final constriction requires MyoJ/Cen2/BCC1, which resides at the very basal end of the BC (BCSC-4). Since onset of constriction coincides with many additional proteins being recruited across the various BC subcomplexes, and that these genes are largely non-essential, it is tempting to assign a function buttressing the unessential process of final constriction. Conversely, these proteins may also be required for recruiting the BC proteins after completion of cell division, which are non-essential in tachyzoites. However, the absence of essential proteins and BC functions complicates the ability to determine the nature of structure function relationships. Assuming these structures and function have been retained under selective pressure, it seems that we have not yet identified the relevant pressures. This also raises the question of how these transitions in composition and function are controlled. We mapped a number of kinases and phosphatases (**Table 1**). Their genome wide CRISPR/Cas9 fitness scores suggest non-essential roles and only HAD2a has a critical function in allowing the BC to progress beyond the midpoint of forming daughters, likely acting on BCC4-MORN1 stability (Engelberg et al., 2016). It is conceivable that the non-essential kinases and phosphatases are redundant and function in parallel pathways, a scenario that deserves serious consideration as the BC has many critical functions to the parasite whose integrity might be worth the cost of dedicating multiple layers of control. With BC composition and architecture largely mapped in both *T. gondii* and *P. falciparum*, the toolboxes are in place to further probe in these open questions to reveal the remaining secrets of the BC.

## Material and Methods

### Parasites and Host Cells

*T. gondii* tachyzoites were maintained and studied in human foreskin fibroblasts (HFF) or studied in VERO cells, as previously described (Roos et al., 1994). Host cells were maintained in DMEM media containing 10% serum. *Toxoplasma* strains RHΔKu80ΔHXGPRT (Huynh and Carruthers, 2009), RH-TaTiΔKu80 (Sheiner et al., 2011), RHΔKu80ΔHXGPRT-Tir1 (Brown et al., 2017) and PrugniaudΔKu80 (PruΔKu80) (Fox et al., 2011) were used as the basis for all mutants in this study. The RHΔMyoI line was generated the same way as the GT1ΔMyoI line described before (Primo et al., 2021). Stable transgenics were obtained by selection under 1 µM pyrimethamine, 25 µg/ml mycophenolic acid (MPA) combined with 50 µg/ml xanthine, or 20 µM chloramphenicol and cloned by limiting dilution.

### Plasmid Cloning and Transgenic Parasite Generation

All oligonucleotides used in this study are listed in **Table S1**. All transgenic lines were cloned by limiting dilution and the genotype validated by diagnostic PCRs (**Figure S2**).

The annotated BTP1 encoding sequence was PCR amplified from RH genomic DNA, including 1.5 kb upstream of the start codon annotated on ToxoDB serving as its own promoter, and was cloned into ptub-YFP-YFP(MCS)/sagCAT (Anderson-White et al., 2011) using *Pme*I and *Avr*II restriction enzymes to replace ptub-YFP. The resulting plasmid was transiently transfected into wild type RH parasites.

To replace the ORF of a gene of interest we first designed CRISPR-Cas9 plasmids that specifically target the 5’ region (and 3’ region in case of FIKK, BCC6 and BCC7) of the respective genes. Oligomers encoding single-guide RNAs were hybridized and ligated into the *Bsa*I-digested pU6-Universal plasmid (Sidik et al., 2016). To facilitate homologous directed repair we PCR-amplified a resistance cassette that drives either DHFR-TSm2m3 (for KOs done in RHΔKu80) or HXGPRT (for KOs done in PRUΔKu80) under the dhfr promoter sequence. Specific integration was facilitated by inclusion of 35 bp flanks on the 5’ and 3’ end of the PCR amplicon, which are homologous to the side of Cas9 double strand break. For transfections; 40 μg of a single Cas9 plasmid (or 20 μg of each in case two Cas9 plasmids were transfected) was mixed with the PCR amplicon, transfected, and were parasites selected with the appropriate drug for proper integration of the resistance marker and deletion of the target gene.

Endogenous tagging of MyoI with a mAID-Myc tag was achieved by PCR amplification of a 2497 bp genomic DNA fragment 3’ of the MyoI translational start and cloned by Gibson assembly into the AAP4-3xMyc-DHFR plasmid (Engelberg et al., 2020) in which the 3xMyc tag was replaced with the mAID-3xMyc coding sequence digested with *Pme*I and *Avr*II. Before transfection the plasmid was linearized with *Avr*II, which cuts within the cloned 3’-region to facilitate homologous recombination in RHΔKu80ΔHXGPRT-Tir1 parasites.

To generate ATc-regulatable CaM (TGGT1_249240) expressing parasites, a 1.3 kb genomic DNA fragment downstream of the ATG codon was amplified using primer pairs CaM-BglII-F/CaM-NotI-R, and cloned by *Bgl*II/*Not*I into an N-terminal cMyc/Ty-epitope tagged plasmid derived from the single homologous recombination plasmid DHFR-tetO7Sag4-Nt-GOI (kindly provided by Dr. Wassim Daher, Université de Montpellier I et II (Morlon-Guyot et al., 2014);). The plasmid was linearized with *Nar*I before transfection.

### Western Blots

Western blot was performed with lysates from parasites treated ± ATc for indicated periods of time. A 12% NuPAGE Bis-Tris (for CaM) (Invitrogen, Thermo Fisher Scientific, USA) was loaded with samples prepared by lysis with 1% SDS in 150 mM NaCl and 50 mM Tris-HCl, pH 8.0, of equal numbers of parasites for each experimental condition. Following SDS-PAGE, proteins were transferred to a PVDF membrane (Bio-Rad, USA) and blocked using 5% milk. Blots were probed with mouse α-tubulin MAb 12G10 (1:2000) and mouse α-Ty (1:500; kindly provided by Dr. Chris de Graffenried, Brown University, USA) followed by probing with horseradish peroxidase (HRP)-conjugated α-mouse (1:10000) (Santa-Cruz Biotech, USA) and detection of signal after chemiluminescent HRP substrate (Millipore, USA) treatment.

### Plaque Assay

T25 culture flasks confluent with HFF cells were inoculated with 100-200 parasites of choice and grown for 7 days. Following incubation, the monolayer was fixed with 100% ethanol for 10 minutes and stained with crystal violet (Roos et al., 1994).

### Indirect Immunofluorescence Assays

For intracellular localization, parasites were inoculated into 6-well plate having coverslips confluent with HFF cells. Following overnight incubation, parasites were fixed with 100% methanol. For extracellular localization, freshly lysed parasites were filtered, pelleted, and resuspended in PBS. Thereafter, parasites were added to poly-L-lysine coated cover-slips and allowed to incubate for 30 min at 4°C prior to fixation with 100% methanol. 1% BSA fraction V in PBS was used as blocking agent.

The following primary antisera were used: α-Myc MAb 9E10 (1:50) (Santa-Cruz Biotech), mouse α-Ty (1:500; kindly provided by Chris de Graffenried, Brown University), rabbit α-IMC3(1-120) [1:2,000 (Anderson-White et al., 2011)], mouse α-GFP (Abgent; 1:500). Guinea pig α-AAP4 [1:200 (Engelberg et al., 2020)]. Alexa 488 (A488) or A594 conjugated goat α-mouse, α-rabbit, or α-guinea pig were used as secondary antibodies (1:500) (Invitrogen). DNA was stained with 4’,6-diamidino-2-phenylindole (DAPI). A Zeiss Axiovert 200 M wide-field fluorescence microscope equipped with a α-Plan-Fluar 100x/1.3 NA and 100x/1.45 NA oil objectives and a Hamamatsu C4742-95 CCD camera was used to collect images, which were deconvolved and adjusted for phase contrast using Volocity software (Perkin Elmer, USA).

### Transmission Electron Microscopy

For basal complex development stage analysis, HFF infected cells were fixed in 4% glutaraldehyde in 0.1 M phosphate buffer pH 7.4 and processed for routine electron microscopy (Ferguson et al., 1999). Briefly, cells were post-fixed in osmium tetroxide and treated with uranyl acetate prior to dehydration in ethanol, treatment with propylene oxide, and embedding in Spurr’s epoxy resin. Thin sections were stained with uranyl acetate and lead citrate prior to examination with a JEOL 1200EX electron microscope.

Basal complex mutant infected cells were prepared for ultrastructural observations by fixation in 2.5% glutaraldehyde in 0.1 mM sodium cacodylate (EMS) and processed as described (Coppens and Joiner, 2003). Ultrathin sections were stained before examination with a Philips CM120 EM (Eindhoven, the Netherlands) under 80 kV.

### GFP-Rab11A Vesicle Assay

VERO cells stably expressing GFP-Rab11A were infected as described before (Romano et al., 2017). In brief, infected cells were fixed in PBS with 0.02% glutaraldehyde (EM grade; EMS) and 4% paraformaldehyde and permeabilized with 0.3% Triton X-100 in PBS for 5 min. Cells were blocked with 3% BSA in PBS followed by incubation in α-TgGRA7 (Coppens et al., 2006) and α-TgNTPase (Bermudes et al., 1994) antiserum, washed with PBS, incubated with Alexa conjugated secondary antibody, washed with PBS and then incubated in 1 μg/ml DAPI in PBS followed by PBS washes. Coverslips were mounted on slides with ProLong antifade mounting solution (Alexa secondary antibodies). Optical z-sections of infected cells with PVs containing 16 parasites to normalize the data for PV size were collected using a Zeiss AxioImager M2 fluorescence microscope equipped with an oil-immersion Zeiss plan Apo 100x/NA1.4 objective and a Hamamatsu ORCA-R2 camera. Optical z-sections were acquired using Volocity software (Quorum Technologies, Puslinch, ON, Canada). Images were deconvolved (confidence limit of 100% and iteration limit of 30-35) and analyzed with a measurement protocol generated in Volocity (Quorum Technologies, UK), which measured objects in the 3D reconstructed volumes of the optical z-slices. In the measurement protocol described in (Romano et al., 2017; Romano et al., 2021), the PV was pinpointed using the fluorescence intensity of TgGRA7 and TgNTPase; the thresholds of the intensity values were set manually by using the outer TgGRA7 PVM staining as the boundary of the PV. GFP-Rab11A foci were identified by percent fluorescence intensity, with the threshold set manually such that there was a clear delineation of GFP-Rab11A puncta in the host cell surrounding the PV, and the GFP-Rab11A foci located within the boundary of the PV were identified. To compare samples to the control, we used a one-way ANOVA with a Tukey’s honest significant difference *post-hoc* test (Graphpad Prism, USA).

### Chronic Infections

Female C57BL/6 mice 3-4 weeks old were i.p. infected with 500 or 2500 mutant or wild type PrugniaudδKu80 strain tachyzoites harvested from an overnight infected HFF monolayer by mechanical, needle lysis, filtration through a 3 μm nylon filter, washed once with, and resuspended in, 1xPBS. Mice were monitored daily for weight and signs of illness. Groups of 4 mice per experiment were used and experiments were repeated twice, unless noted otherwise. Between 3 and 4 weeks post infection, mice were sacrificed through CO_2_ inhalation, the brains harvested and the cysts enriched and quantified following published methods (Mordue et al., 2007). In brief, brains were ground up in 1300 μl 1xPBS using mortar and pestle. A 250 μl aliquot of the slurry from two pooled mice brains was subsequently passed five times each through 16G, 18G, 20G, and 23G needles. Samples were fixed by adding 150 μl 3% formaldehyde in 1xPBS for 20 min at RT, spun for 5 min at 3000xg, and quenched with 150 μl 0.1 M glycine in 1xPBS for 5 min followed by another spin and combined blocking and permeabilization using 150 μl BP-mix (3% BSA in 1xPBS in 0.2% TX-100 in 1xPBS) for 1 hr at RT, or overnight at 4°C. Following a spin, 150 μl of Fluorescein-conjugated *Dolichos bifloris* agglutin lectin (Fluorescein-DBA; 1:3000; Vector Laboratories, USA) in BP-mix was incubated for 1 hr at RT. After three washes with 150 μl BP-mix, 5 μl of the brain pellet was spread and mounted on three different slides, which were all counted by fluorescence microscopy. The total cyst number multiplied by 26 represents the total number of cysts/single brain. Animal protocols were reviewed and approved by the Boston College IACUC with protocol number 2018-001.

### Rosette Assay

Directly A488 conjugated T41E5 α-SAG1 antibody (Couvreur et al., 1988), kindly provided by Dr. Jean-François Dubremetz, was used to stain 4% PFA fixed vacuoles permeabilized with 0.25% TX-100 (both in 1xPBS) 30 hr infected in HFF cells. Vacuoles containing at least 16-parasites were visualized by fluorescence microscopy and enumerated for random or rosette organization of the tachyzoites. Three biological replicates were performed and at least 100 vacuoles per sample were counted.

## Data Availability Statement

The original contributions presented in the study are included in the article/**Supplementary Material**. Further inquiries can be directed to the corresponding authors.

## Ethics Statement

Animal protocols were reviewed and approved by the Boston College IACUC with protocol number 2018-001.

## Author Contributions

M-JG designed experiments, generated schematics, wrote the manuscript, acquired funding; DF performed TEM for the BC development cycle; SS generated the CaM-cKD line and evaluated the genotype and phenotype; JDR performed host Rab11A-GFP accumulation assays; SC generated BC gene knock out strains in the Pru line, performed chronic infection experiments; VP generated the MyoI-KO parasite line and evaluated phenotype; CM generated MyoI-cKD parasite line and performed the rosette and plaque formation assays on BC mutant strains; IC performed TEM on BC mutant strains; KE designed experiments, generated BC knock out strains in the RH line, edited the manuscript, acquired funding. All authors proofread the manuscript. All authors contributed to the article and approved the submitted version.

## Funding

This study was supported by National Science Foundation (NSF) Major Research Instrumentation grant 1626072, National Institute of Health grants AI107475, AI117241, AI110690, AI144856, AI128136, and AI152387, an American Heart Association post-doctoral fellowship grant 17POST33670577, a Knights Templar Eye Foundation early career starter grant and an Ignite Program award through Boston College. The funders had no role in study design, data collection and analysis, decision to publish, or preparation of the manuscript.

## Conflict of Interest

The authors declare that the research was conducted in the absence of any commercial or financial relationships that could be construed as a potential conflict of interest.

The reviewer SB declared a past co-authorship/collaboration with one of the authors with the author IC to the handling Editor.

## Publisher’s Note

All claims expressed in this article are solely those of the authors and do not necessarily represent those of their affiliated organizations, or those of the publisher, the editors and the reviewers. Any product that may be evaluated in this article, or claim that may be made by its manufacturer, is not guaranteed or endorsed by the publisher.
