# Supplementary material for: Toxoplasma gondii’s Basal Complex: The Other Apicomplexan Business End Is Multifunctional
Source: Front Cell Infect Microbiol. 2022 Apr 29;12:882166. doi: 10.3389/fcimb.2022.882166 (PMC9103881; doi:10.3389/fcimb.2022.882166)
Supplement: Supplementary file 1 [file DataSheet_1.docx]

**Supplementary Material**

**Table S1. Primers used for cloning and PCR validation of transgenic parasites.**

**Table S2. Expected product sizes for the BC mutant validation diagnostic PCR reactions.**

**Table S3. Plaque assay raw and processed data counts for the BC mutants.**

**Table S4. GFP-Rab11A imaging data collection and analysis for select BC mutants.** See [1] for detailed description of data collection.

**Supplementary Figure S1. Conditional knock-down of calmodulin and MyoI.**

**A.** Western blot analysis displaying the kinetics of CaM depletion upon ATc treatment. Ty highlights CaM, whereas tubulin is used as loading control.

**B.** Depletion of CaM upon ATc addition for 24 hrs does not have an appreciable effect on the morphology of either mother or daughter buds. AAP4 stains the apical annuli and its position marks the apical end or parasites thereby permitting the inference of BC localization at the other end of the cytoskeleton scaffolds marked by IMC3; DAPI marks the DNA.

**C.** Prolonged CaM depletion is lethal as illustrated by plaque assays for 7 days. TaTiΔKu80 is the parent line.

**D.** IFA analysis demonstrating MyoI-mAID-Myc is depleted after 24 hrs of IAA treatment. Note the cytoplasmic bridge connection organizing the parasites in the same vacuole is disrupted. Myc marks MyoI; cytoskeleton scaffolds marked by IMC3; DAPI marks the DNA. Basal ends of the IMC cytoskeleton are marked with asterisks.

**Supplementary Figure S2. PCR validation of transgenic lines generated in this study.**

**A.** Calmodulin conditional knock-down line. cKD, conditional knock-down; locus, the original locus across the targeted site of insertion. int.1, integration primer pair #1; int. 2, integration primer pair #2. DNA sizes indicated on the left of gel.

**B.** MyoI conditional knock-down line. WT, RH-Tir1; int., integration primer. DNA sizes indicated on the left of gel.

**C. Knock-out lines, as indicated.** select., drug selectable marker, which was DHFR-TS for RH genotype lines, and HXGPRT for Prugniaud (Pru) genotype lines. KO, knock-out, Pa, parent line; 5’, 5’-flank of the integration site; 3’, 3’-flank of the integration site; ORF, open reading frame of the targeted gene; locus, PCR across the integrated drug selectable marker. DNA sizes indicated on the left of all gels. Note that the ‘locus’ PCR may not generate a band if the targeted genomic area is larger than the amplifiable size (typically, >5 kb is challenging to amplify). The predicted sizes of all PCR reactions is provided in **Table S2**.

**Supplementary Figure S3. Plaque assays of transgenic BC protein knock-out and knock-down lines.**

**A.** Representative plaque assays for each cell line.

**B.** Quantified plaque numbers, normalized against the inoculum. n=3, average +std. No significant differences were detected between any of the strains (student’s *t*-test). Source data available in **Table S3**.

**Supplementary Figure S4.** Prugniaud strain BC protein knock-out and control parasites were i.p. injected in female C57/BL6 mice. Brains were harvested 3-4 weeks post infection and cysts purified from two pooled mice brains and enumerated following DBA staining of the cyst wall. n.d. = not done.

**Supplementary references**

[1] J.D. Romano, E.J. Hartman, and I. Coppens, Quantitative Fluorescence Microscopy for Detecting Mammalian Rab Vesicles within the Parasitophorous Vacuole of the Human Pathogen Toxoplasma gondii. Methods Mol Biol 2293 (2021) 295-305.
