# Supplementary figures and images for: Toxoplasma gondii’s Basal Complex: The Other Apicomplexan Business End Is Multifunctional
Source: Front Cell Infect Microbiol. 2022 Apr 29;12:882166. doi: 10.3389/fcimb.2022.882166 (PMC9103881; doi:10.3389/fcimb.2022.882166)

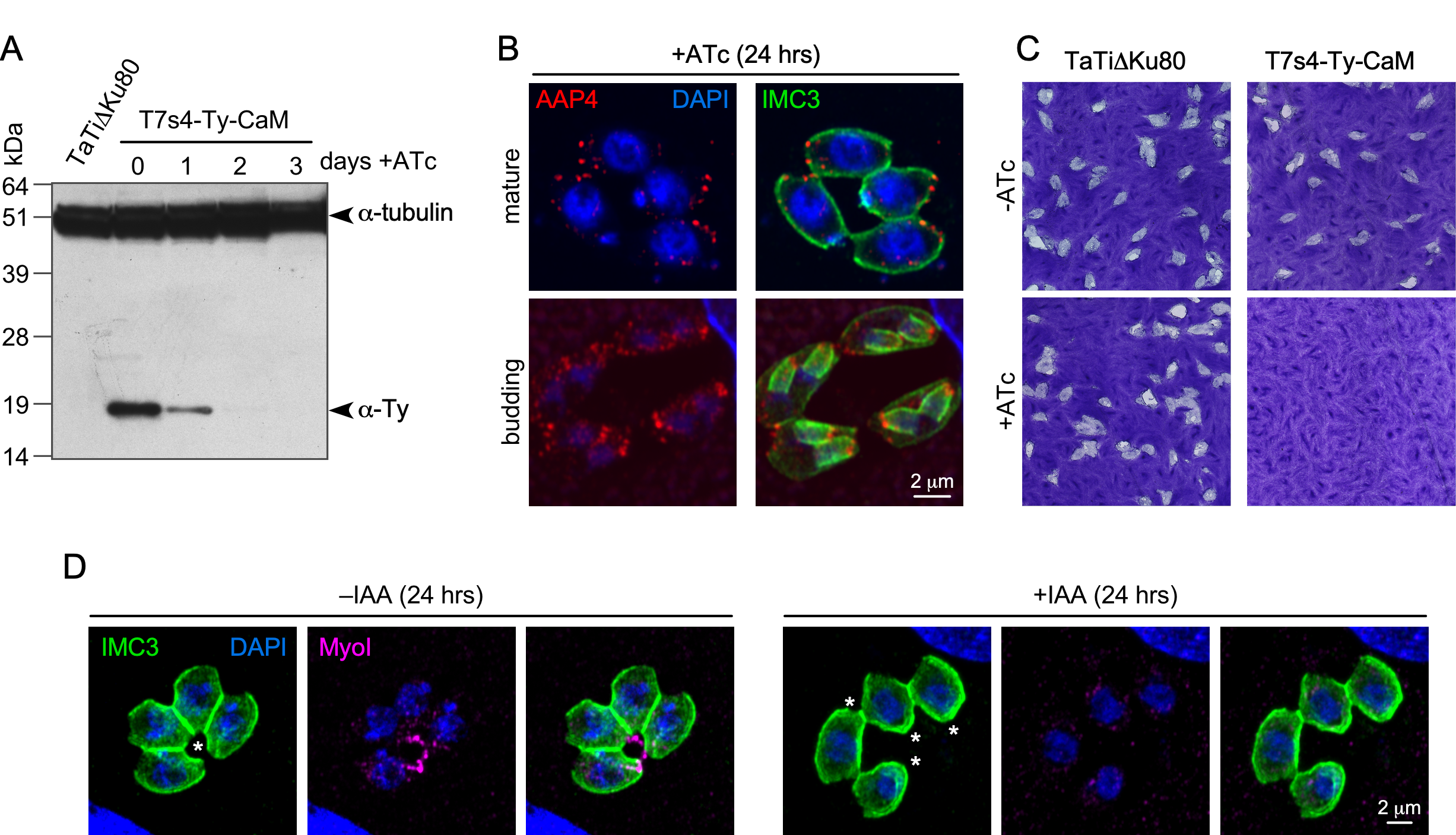

Supplement: Supplementary file 2 [file Image_1.tif]

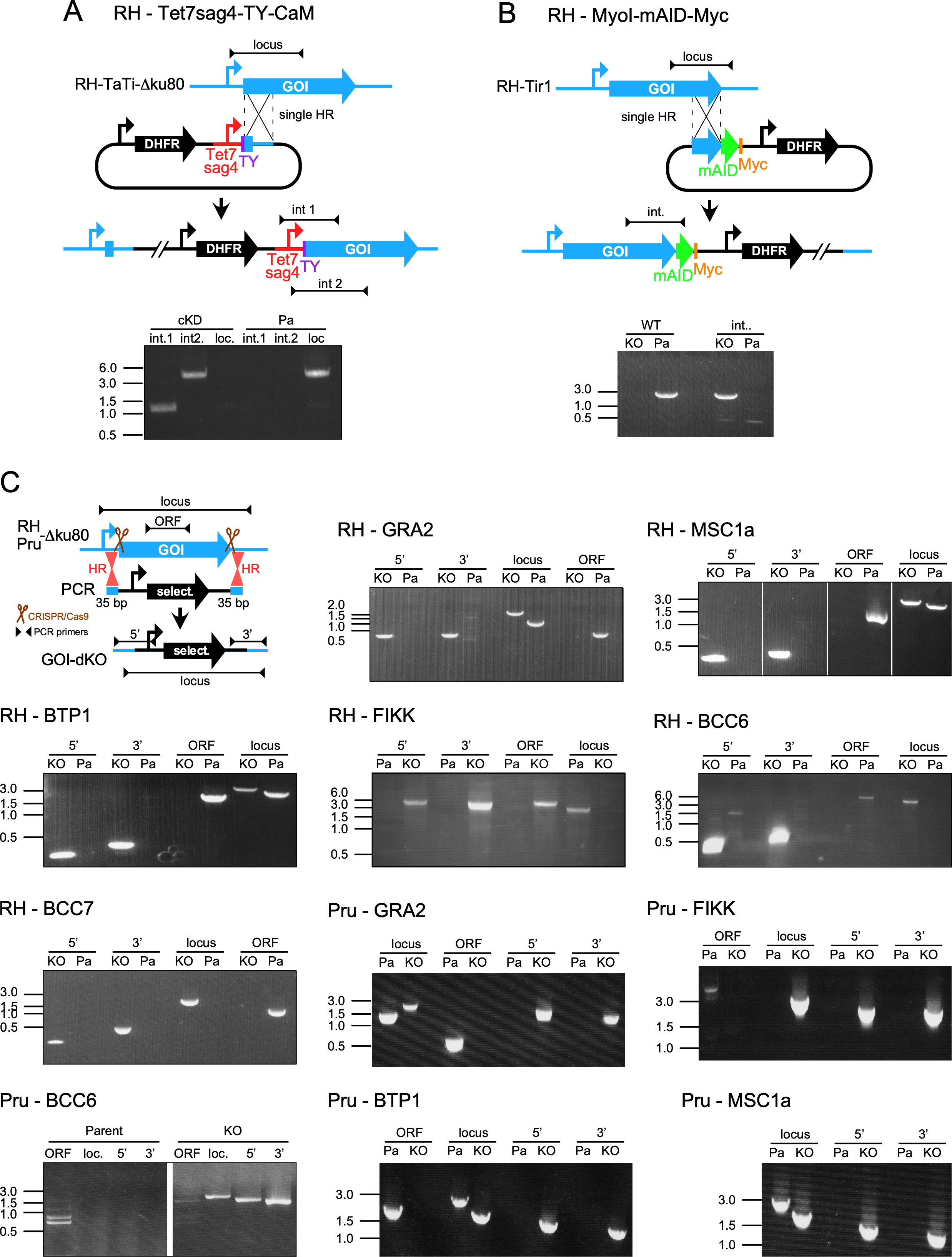

Supplement: Supplementary file 3 [file Image_2.tif]

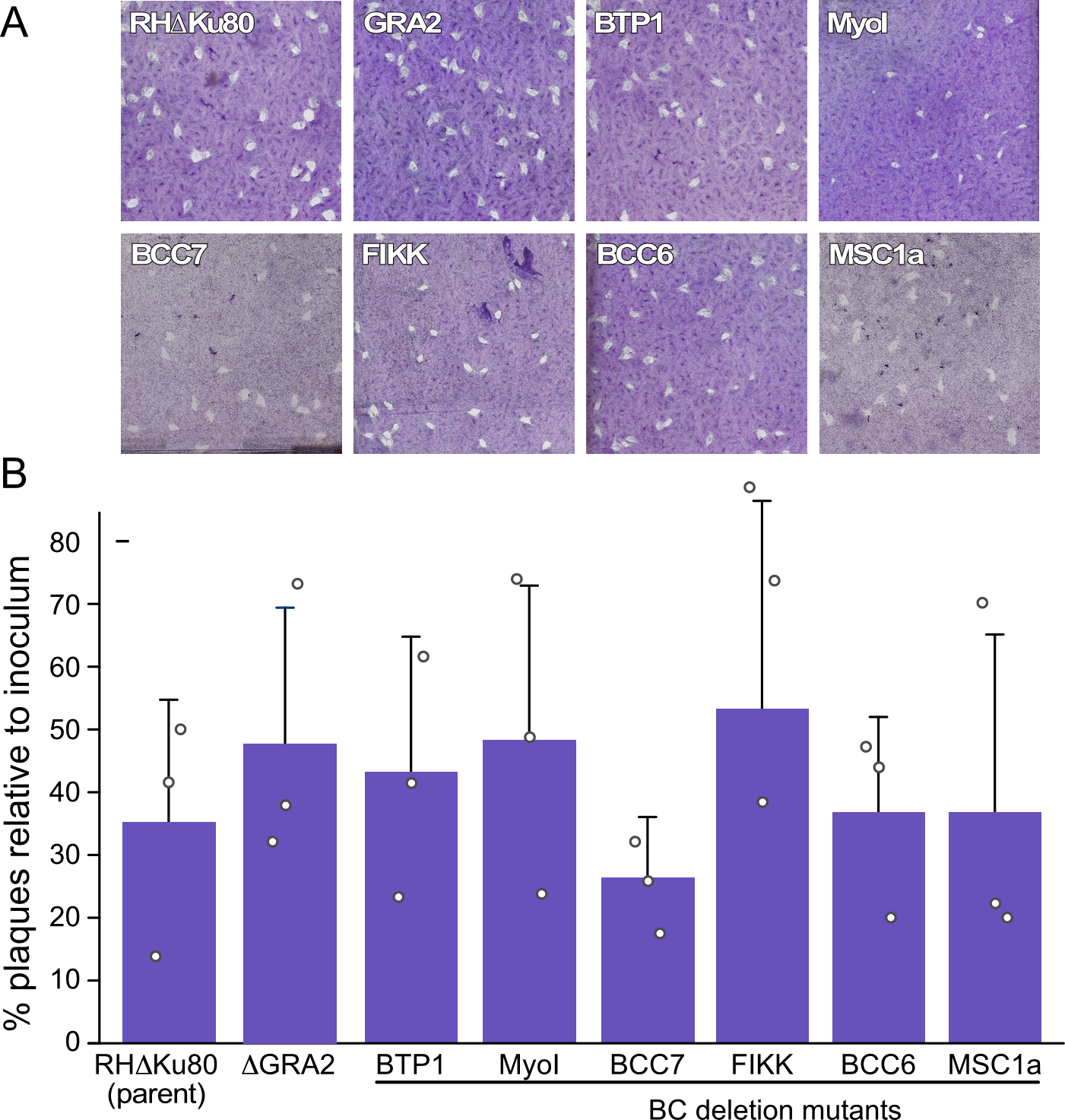

Supplement: Supplementary file 4 [file Image_3.tif]

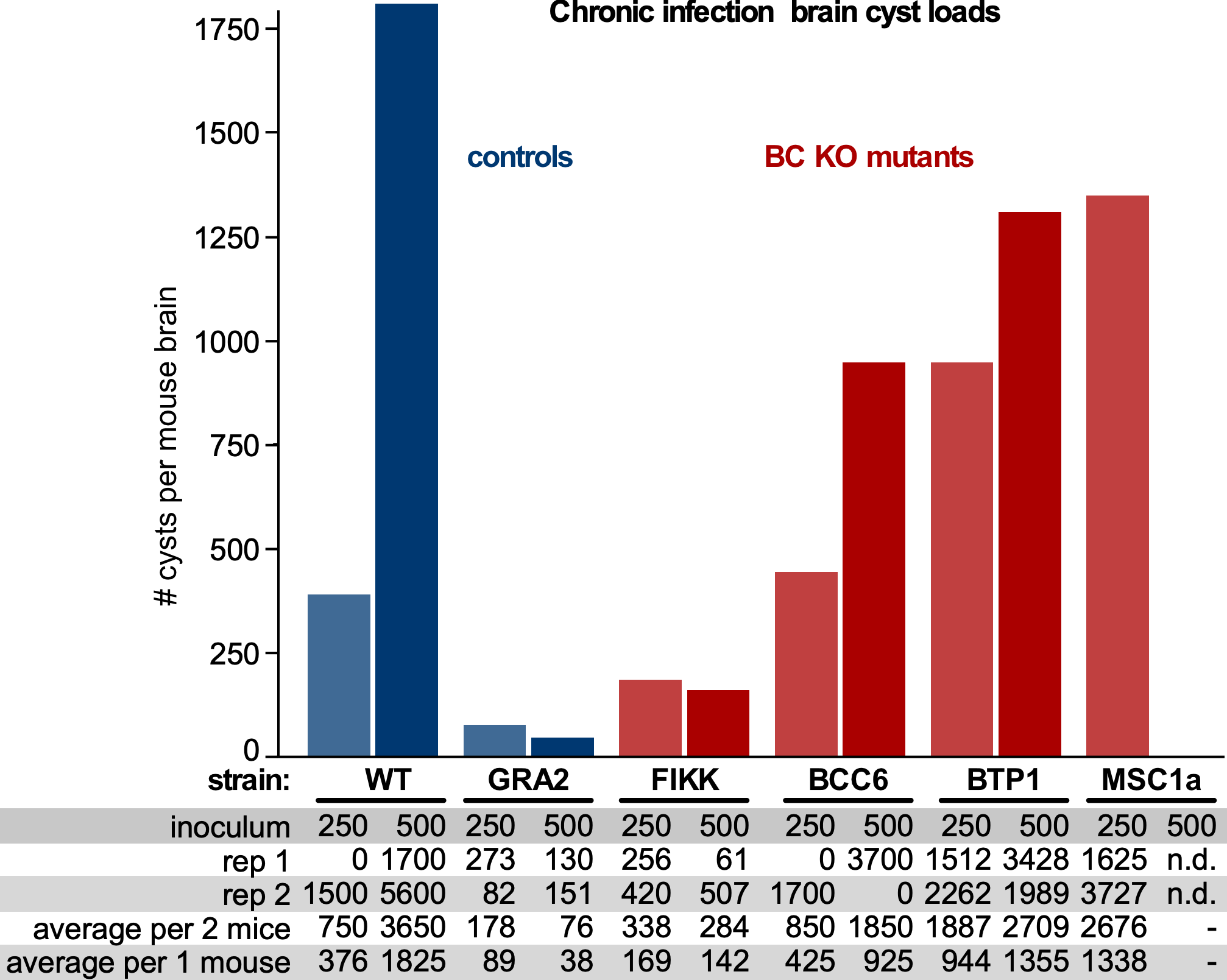

Supplement: Supplementary file 5 [file Image_4.tif]
